# DSCA-HLAII: A dual-stream cross-attention model for predicting peptide–HLA class II interaction and presentation

**DOI:** 10.1371/journal.pcbi.1013836

**Published:** 2026-01-02

**Authors:** Ke Yan, Hongjun Yu, Shutao Chen, Alexey K. Shaytan, Bin Liu, Youyu Wang

**Affiliations:** 1 School of Computer Science and Technology, Beijing Institute of Technology, Beijing, China; 2 Zhongguancun Academy, Beijing, China; 3 Department of Biology, Lomonosov Moscow State University, Moscow, Russia; 4 Centre for Biomedical Research and Technology, AI and Digital Sciences Institute, Faculty of Computer Science, HSE University, Moscow, Russia; 5 SMBU-MSU-BIT Joint Laboratory on Bioinformatics and Engineering Biology, Shenzhen MSU-BIT University, Shenzhen, Guangdong, China; 6 Department of Thoracic Surgery, Sichuan Academy of Medical Sciences and Sichuan Provincial People’s Hospital, University of Electronic Science and Technology of China, Chengdu, Sichuan Province, China; Xinjiang Technical Institute of Physics and Chemistry, CHINA

## Abstract

**Motivation:**

The interaction between peptides and human leukocyte antigen class II (HLA-II) molecules plays a pivotal role in adaptive immune responses, as HLA-II mediates the recognition of exogenous antigens and initiates T cell activation through peptide presentation. Accurate prediction of peptide-HLA-II binding serves as a cornerstone for deciphering cellular immune responses, and is essential for guiding the optimization of antibody therapeutics. Researchers have developed several computational approaches to identify peptide-HLA-II interaction and presentation. However, most computational approaches exhibit inconsistent predictive performance, poor generalization ability and limited biological interpretability.

**Results:**

In this study, we present DSCA-HLAII, a novel predictive framework for peptide-HLA-II interactions and presentation based on a dual-stream cross-attention architecture. The framework proposes a dual-stream cross-attention (DSCA) mechanism to integrate pre-trained semantic embedding ESMC with sequence-level ONE-HOT features. The DSCA mechanism effectively models the interaction dynamics between peptides and HLA-II molecules, enabling the precise identification of key binding sites. Experimental results demonstrate that DSCA-HLAII consistently surpasses existing state-of-the-art approaches, demonstrating high accuracy and robustness in predicting peptide-HLA-II interactions and presentation. We further demonstrate the capability of DSCA-HLAII for predicting peptide binding cores and assessing antibody immunogenicity, which is expected to advance artificial intelligence-based peptide drug discovery.

## Author summary

This paper proposes a novel predictive framework for peptide-HLA-II interactions and presentation based on a dual-stream cross-attention architecture (DSCA-HLAII). DSCA-HLAII is a unified predictive framework that integrates sequence-based ONE-HOT features with pre-trained semantic embeddings (ESMC) to construct a comprehensive hybrid representation of peptides and full-length HLA-II sequences. By introducing a Dual-Stream Cross-Attention (DSCA) module, the model enables fine-grained characterization of peptide–HLA-II interactions and assigns differential scores across sequence positions, thereby improving the identification of critical binding sites and enhancing generalization. DSCA-HLAII simultaneously predicts peptide presentation probability and binding core location, and further supports systematic assessment of antibody immunogenicity risk. Extensive experiments on multiple warm-start test datasets and cold-start test datasets demonstrate that DSCA-HLAII surpasses existing state-of-the-art methods in both accuracy and robustness. Additionally, a publicly accessible web server (http://bliulab.net/DSCA-HLAII) has been established to facilitate practical application.

## 1. Introduction

Major histocompatibility complex (MHC) molecules hold a central position in adaptive immunity. In humans, the MHC molecules are known as human leukocyte antigens (HLAs). HLAs are divided into two primary classes: class I (HLA-I) and class II (HLA-II). Typically, HLA-II molecules are divided by three highly polymorphic loci: HLA-DR, HLA-DP, and HLA-DQ [1]. These isotypes form heterodimers composed of an *α* chain and a *β* chain protein [2]. Exogenous antigenic peptides are loaded onto HLA-II molecules, and the resulting complexes are transported to the cell surface for presentation. The binding affinity between a peptide and an HLA-II molecule is primarily determined by interactions within the peptide’s binding core [3].These complexes are recognized by CD4 ⁺ helper T cells, thereby inducing both humoral and cellular immune responses [4,5]. Therefore, the specificity and strength of peptide–HLA-II interactions directly influence which peptides can be effectively presented and recognized. Due to the highly complex and polymorphic nature of HLA-II molecules, accurately predicting peptide presentation and assessing their immunogenic potential remain critical challenges in immunology research and clinical immunotherapy.

In previous studies, researchers employed *in vitro* experimental techniques such as mass spectrometry and immunopeptidomics to investigate the binding and presentation mechanisms of peptides by HLA-II molecules [6–8]. These efforts have provided abundant reliable data and prior knowledge for subsequent research. However, *in vitro* experiments are costly and time-consuming, and thus cannot achieve comprehensive coverage of such interactions [9]. To overcome these limitations, a series of pan-specific computational tools have been developed and applied to the prediction of peptide-HLA-II binding and presentation, becoming essential methods in this field [10–12]. These pan-specific tools construct computational models to predict peptide–HLA-II interactions for both known alleles and previously unseen alleles [13]. The strategy effectively alleviates the constraints caused by limited data for certain alleles and offers broader applicability. Racle et al. proposed a probabilistic method, MixMHC2pred, which employs the Expectation–Maximization (EM) algorithm to learn probabilistic graphical models of multiple motifs [14]. Nilsson et al. introduced NetMHCIIpan, a multilayer perceptron (MLP)-based model for predicting peptide–HLA-II binding and presentation [15]. Thrift et al., leveraging prior knowledge from AlphaFold2-multimer, developed a graph convolutional network model Graph-pMHC that explicitly captures peptide–HLA-II interactions [16,17]. Wang et al. constructed TripHLApan, a deep neural network integrating gated recurrent units (GRUs) and attention mechanisms to predict peptide–HLA-II binding [18]. Chang et al. designed CapHLA, a hybrid network to jointly predict peptide–HLA-II binding affinity and presentation probability [19].

Despite the remarkable progress made in peptide-HLA-II interactions and presentation studies in recent years, there remain several disadvantages [20–22]. Firstly, previous studies typically represented HLA-II molecules by extracting specific residues from resolved allele structures as pseudo sequences [23,24]. Peptide sequences and pseudo sequences of HLA-II molecules were then represented by hand-crafted features, such as amino acid composition and physicochemical properties [3,23,25–29]. However, such representations fail to capture the contextual dependencies and global semantic information across residues, which are essential for accurate identifying peptide-HLA-II binding [30–34]. Secondly, the key sites directly influence the interactions between peptides and HLA-II molecules. These key sites are typically located in specific regions, such as the peptide binding cores and certain pockets of HLA-II molecules [35]. Accurately identifying key sites such as the binding core not only reflects whether the model has adequately captured the underlying mechanism of peptide–HLA-II presentation, but also serves as an indicator of its interpretability and biological reliability. Moreover, accurate identification of the binding core facilitates downstream tasks such as CD4 ⁺ T cell recognition. Nevertheless, existing approaches often treat all residues uniformly and fail to explicitly capture the key sites which are essential for accurately characterizing the interaction between peptides and HLA-II molecules [36]. Thirdly, despite their satisfactory performance with known alleles, their models fail to generalize effectively, exhibiting poor performance for identifying unknown alleles.

To address the challenges, we propose DSCA-HLAII, an end-to-end deep learning framework based on a Dual-Stream Cross-Attention (DSCA) mechanism, designed to provide a robust and high-performance solution for predicting peptide–HLA-II presentation probabilities. The contributions of the proposed method are as follows:

(1) DSCA-HLAII integrates sequence-based ONE-HOT features [37,38] with pre-trained semantic embeddings ESMC, which is derived from large-scale unsupervised data [39]. By fusing multi-modal features into a hybrid representation of both peptide sequences and complete sequences of HLA-II chains, DSCA-HLAII achieves a more comprehensive representational space than that provided by hand-crafted features.
(2) DSCA-HLAII introduces the DSCA module to facilitate more precise modeling of peptide–HLA-II interactions and allow differential weighting across sequence sites, thereby enhancing the identification of key sites. By partially mimicking the biological interaction process, the DSCA module enhances the model’s generalization ability.
(3) The ONE-HOT features capture the residue-level information, whereas the ESMC features encode the semantic and structural characteristics of the sequences. The DSCA mechanism captures the interaction information between peptides and HLA-II molecules. By leveraging fused features and integrating the DSCA mechanism, we further enhance the model’s predictive capability, particularly improving its generalization performance on unknown alleles.
(4) DSCA-HLAII is capable of jointly predicting peptide presentation probability and binding core position, while also providing a systematic assessment of antibody immunogenicity risk. Comparative experiments across multiple independent test datasets demonstrate that DSCA-HLAII outperforms current state-of-the-art methods in both predictive accuracy and generalization performance, highlighting its potential value for peptide–HLA-II research and immunological applications. Finally, we developed a user-friendly web server, which has been made publicly accessible at http://bliulab.net/DSCA-HLAII.

## 2. Results

### 2.1 Comparison with the existing baseline methods on the warm-start test dataset

In this study, we employed a 5-fold cross-validation strategy to compare the performance of DSCA-HLAII with several baseline methods on the warm-start test dataset, including NetMHCIIpan-4.3 [15], CapHLA [19], TripHLApan [18], Graph-pMHC [16] and MixMHCIIPred [14].

**Fig 1:**
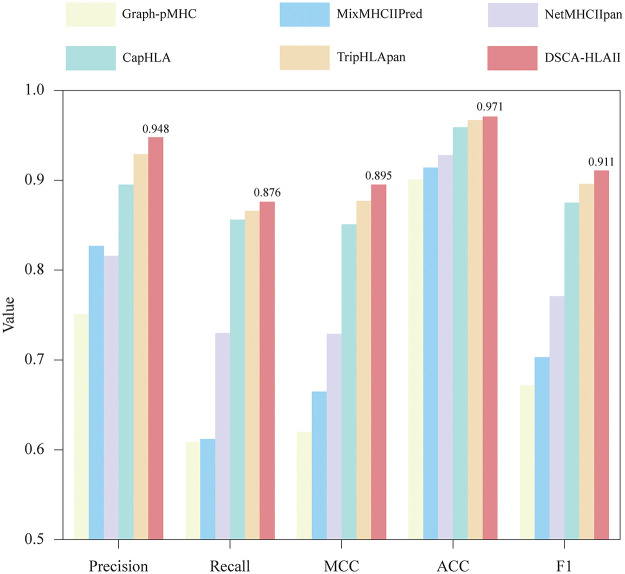
Performance comparison on the warm-start test dataset in terms of Precision, Recall, MCC, ACC and F1.

In the warm-start test dataset, all HLA-II alleles appeared in the benchmark dataset, aiming to evaluate the predictive performance on previously seen alleles. As shown in Table 1 and Fig 1, DSCA-HLAII demonstrates superior performance than the five baseline methods in terms of AUROC, AUPR, PCC, Precision, Recall, MCC, ACC, and F1. To evaluate whether the observed performance differences were statistically significant, we conducted a bootstrap analysis on the warm-start test dataset. Compared to the strongest baseline, TripHLApan, DSCA-HLAII achieved an AUROC improvement of 0.0038, with a 95% bootstrap confidence interval of 0.0033–0.0043, indicating that the improvement is statistically significant (p < 0.001). On the warm-start test dataset, DSCA-HLAII demonstrates a genuine and statistically significant improvement over the strongest baseline, TripHLApan, confirming the effectiveness of the model in enhancing predictive performance.

**Table 1:** Performance comparison on the warm-start test dataset in terms of AUROC, AUPR and PCC.

	AUROC	AUPR	PCC
Graph-pMHC	0.891	0.747	0.291
MixMHCIIPred	0.845	0.706	0.628
NetMHCIIpan	0.889	0.811	0.751
CapHLA	0.970	0.926	0.795
TripHLApan	0.984	0.955	0.889
DSCA-HLAII	**0.988**	**0.963**	**0.909**

The results show that (1) The proposed method achieves a strong linear correlation between the predicted probabilities and the ground truth labels, as evidenced by a high PCC value, confirming its reliability in generating probability estimates. (2) Unlike MixMHCIIPred and NetMHCIIpan, which extract features from HLA-II pseudo-sequences, or Graph-pMHC, which uses limited inputs, our method leverages ESMC to build intrinsic biological representations from complete chain sequences. By operating on the entire chain sequence, ESMC directly learns to decipher the intrinsic information that encode both structure and function. This provides a more comprehensive representation than pseudo sequence which offers only an incomplete sequence information. (3) Compared with CapHLA and TripHLApan adopt relatively simple network architectures and lack explicit modeling of the antigen presentation process, the proposed method utilized DSCA mechanism enables the capture of contextual information and the identification of key sites, thereby achieving explicit modeling of the interaction process between peptides and HLA-II molecules. Therefore, DSCA-HLAII achieves superior accuracy and robustness against existing state-of-the-art methods in peptide–HLA-II presentation prediction.

**Fig 2:**
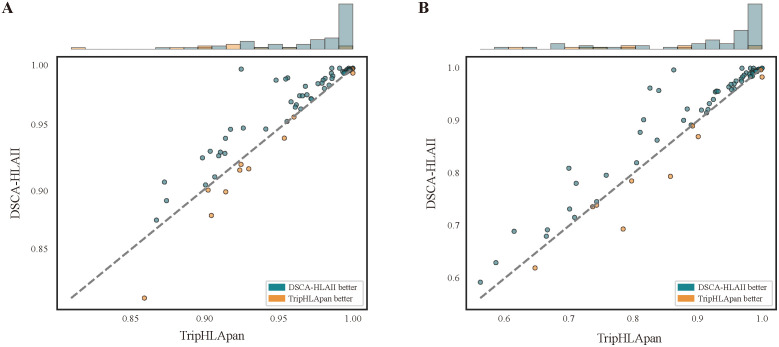
Performance comparison between DSCA-HLAII and TripHLApan across different alleles on the warm-start test dataset. A: The results based on AUROC. B: The results based on AUPR.

To further assess the predictive performance of our method across different HLA-II alleles, we conducted a per-allele comparison between DSCA-HLAII and TripHLApan [18] on the warm-start test dataset. In terms of best AUROC and AUPR, TripHLApan takes the second place in Table 1 by integrating triple coding matrix and transfer learning strategy. As illustrated by the allele distribution in Fig 2, DSCA-HLAII demonstrates superior performance over TripHLApan on 63 alleles. For example, for the allele DRA*01:01-DRB1*08:02, DSCA-HLAII achieves AUROC and AUPR scores of 0.926 and 0.796, whereas TripHLApan attains only 0.898 and 0.758. This performance gap can be partially attributed to the fact that TripHLApan relies solely on AAindex, Blosum62, and integer encoding as input features, while the number of samples available for this allele in the benchmark dataset is limited to 4,861. Consequently, the model struggles to capture effective peptide–HLA-II interaction patterns from such sparse data. In contrast, DSCA-HLAII leverages a fused representation of ONE-HOT and ESMC, enabling it to extract deeper biological information from large-scale sequence data and thereby improving predictive accuracy even for alleles with limited sample sizes. Therefore, DSCA-HLAII achieves consistently strong predictive performance across a wide range of alleles, rather than being effective only for a subset, underscoring its robust generalization capability.

### 2.2 Comparison with existing baseline methods on the cold-start test dataset

**Fig 3:**
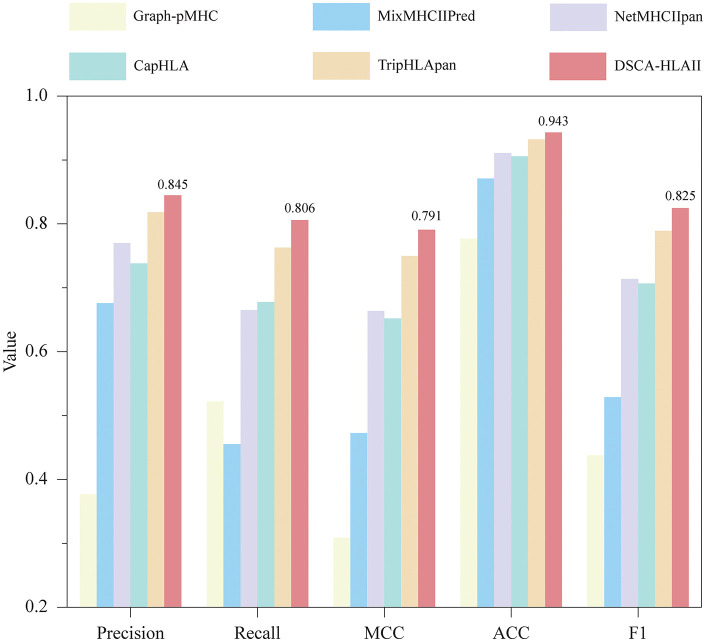
Performance comparison on the cold-start test dataset in terms of Precision, Recall, MCC, ACC and F1.

To evaluate the generalization capability of the model on unseen alleles, we compared the performance of DSCA-HLAII with several baseline methods on the cold-start test dataset. The methods included NetMHCIIpan-4.3 [15], CapHLA [19], TripHLApan [18], Graph-pMHC [16] and MixMHCIIPred [14]. In the cold-start test dataset, all HLA-II alleles are entirely absent from the benchmark dataset, specifically designed to assess the model’s generalization performance on novel alleles. The experimental results are presented in Table 2 and Fig 3. Similarly, we performed a bootstrap analysis on the cold-start test dataset, which confirmed that DSCA-HLAII achieved a significant improvement over TripHLApan, with an AUROC increase of 0.0138 and a 95% bootstrap confidence interval of 0.0070–0.0202 (p < 0.001). On the cold-start test dataset, DSCA-HLAII similarly demonstrates a genuine and statistically significant improvement in performance.

**Table 2:** Performance comparison on the cold-start test dataset in terms of AUROC, AUPR and PCC.

	AUROC	AUPR	PCC
Graph-pMHC	0.773 (-0.118)	0.474 (-0.273)	0.236 (-0.055)
MixMHCIIPred	0.806 (-0.039)	0.586 (-0.120)	0.517 (-0.111)
NetMHCIIpan	0.882 (-0.007)	0.654 (-0.157)	0.545 (-0.206)
CapHLA	0.890 (-0.08)	0.762 (-0.164)	0.644 (-0.151)
TripHLApan	0.949 (-0.035)	0.863 (-0.092)	0.769 (-0.12)
DSCA-HLAII	**0.963 (-0.025)**	**0.894 (-0.069)**	**0.803 (-0.106)**

The results indicate that: (1) On the cold-start test dataset, DSCA-HLAII outperforms all other methods across multiple metrics, including Precision, AUROC, and AUPR. Even when the dataset contains entirely novel alleles, our method maintains high predictive performance. (2) We further quantified the performance differences of each method between the cold-start and warm-start test datasets with the detailed results (AUROC, AUPR and PCC) shown in parentheses in Table 2. DSCA-HLAII proved to be the most robust method, exhibiting only a minimal performance degradation in the cold-start setting, in contrast to the significant declines seen in other methods. These findings indicate that DSCA-HLAII, based on the DSCA mechanism, maintains robust predictive performance when encountering unseen alleles.

**Fig 4:**
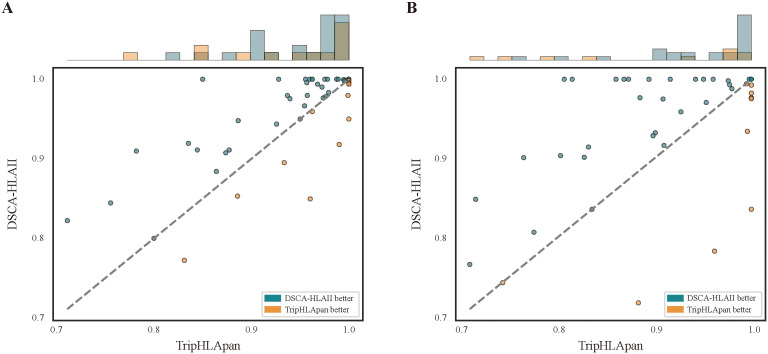
Performance comparison between DSCA-HLAII and TripHLApan across different alleles on the cold-start test dataset. A: The results based on AUROC. (B)The results based on AUPR.

We further compared the performance of DSCA-HLAII and TripHLApan on individual alleles within the cold-start test dataset. According to the allele distribution shown in Fig 4, DSCA-HLAII outperforms TripHLApan on 34 alleles. For example, for DRA*01:01-DRB1*14:02, DSCA-HLAII achieves AUROC and AUPR values of 0.910 and 0.799, respectively, whereas TripHLApan attains only 0.782 and 0.615. This indicates that TripHLApan experiences a substantial performance decline when predicting entirely novel alleles. In contrast, DSCA-HLAII maintains superior predictive capability even on previously unseen alleles.

In summary, these results demonstrate that the incorporation of ESMC features enables DSCA-HLAII to achieve a more comprehensive and fine-grained representation of sequence semantics, structural characteristics, and functional attributes, even in the context of previously unobserved alleles. Furthermore, the DSCA mechanism facilitates the efficient extraction of presentation-relevant information through explicit modeling and precise identification of key residues. Therefore, the test results demonstrate that DSCA-HLAII achieves superior accuracy and robustness across all metrics compared to the baseline methods.

### 2.3 Ablation analysis of DSCA-HLAII

In this section, we conducted ablation experiments on the warm-start test dataset to evaluate the contributions of the fused ESMC–ONE-HOT representations and the DSCA module. The results are summarized in Table 3. Compared with Model 1, it is evident that the model can learn comprehensive semantic, structural, and functional information from ESMC, thereby enhancing predictive accuracy. Compared with Model 2, the fused representation of the complete sequences of HLA-II chains using ESMC and ONE-HOT outperforms the ONE-HOT representation of pseudo sequences. These results indicate that representing HLA-II molecules with complete sequences of chains and employing the fused representation further improves model performance. The fused representation incorporates external knowledge, such as semantic and structural information, augmenting the representation space of peptides and HLA-II molecules and enhancing the predictive capability of DSCA-HLAII. In Model 3 and Model 4, we employed other pretrained protein language models, ProtBert [40] and ProtT5 [41], respectively, to compare the performance differences between different embeddings. Compared with Model 3 and Model 4, the model using ESMC demonstrates superior performance on the warm-start test dataset. ProtBert and ProtT5 primarily learn the statistical patterns of amino acid sequences through self-supervised sequence prediction. In contrast, ESMC integrates both sequence and structural information in its training objectives. As a result, ESMC captures spatial structures, functional sites, and other biologically relevant features more effectively, which are highly relevant to the peptide–HLA-II binding mechanism. Therefore, we attribute the advantages of ESMC over other models primarily to this capability.

**Table 3:** The ablation analysis in the DSCA on the warm-start test dataset.

Method	Repr^a^	Repr^b^	Repr^c^	Repr^d^	Repr^e^	CA^a^	CA^b^	AUROC	AUPR
**baseline**	√					√		**0.988**	**0.963**
**Model1**		√				√		0.981	0.945
**Model2**			√			√		0.980	0.955
**Model3**				√		√		0.981	0.947
**Model4**					√	√		0.982	0.951
**Model5**	√						√	0.788	0.454

Note: Repr^a^ denotes the representation of HLA-II molecules using the fused information of ESMC and ONE-HOT from the complete sequences of chains. Repr^b^ denotes the representation using only ONE-HOT from the complete sequences of chains. Repr^c^ denotes the representation using ONE-HOT from pseudo sequences. Repr^d^ denotes the representation of HLA-II molecules using the fused information of ProtBert and ONE-HOT from the complete sequences of chains. Repr^e^ denotes the representation of HLA-II molecules using the fused information of ProtT5 and ONE-HOT from the complete sequences of chains. CA^a^ indicates that the attention module employs the DSCA mechanism, whereas CA^b^ indicates that the attention module employs the conventional cross-attention mechanism.

Compared with Model 5, the DSCA mechanism demonstrates superior performance over the conventional cross-attention mechanism, confirming its advantage and applicability in modeling peptide-HLA-II interactions and improving both representational power and predictive performance. We attribute this improvement to the dual-stream and cross-stream attention design, as well as the Query–Key–Value construction based on global and local features, all of which are absent in the conventional cross-attention mechanism.

In addition, the longitudinal comparison of the experimental results shows that both ESMC and the DSCA mechanism contribute positively to model performance. By incorporating the rich representational information provided by ESMC, the performance in terms of AUROC and AUPR improves from 0.981 and 0.945 in Model 1 to 0.988 and 0.963, respectively. Through the effective modeling enabled by the DSCA mechanism, the AUROC and AUPR similarly increase from 0.788 and 0.454 in Model 1 to 0.988 and 0.963. These results indicate that DSCA-HLAII, supported by a well-designed modeling strategy and architecture, achieves superior performance on the peptide–HLA-II presentation task by further integrating the rich semantic and structural information encoded by ESMC.

Overall, the experimental results demonstrate that the combination of fused representations and the DSCA module synergistically promotes accurate modeling of peptide–HLA-II interactions and presentation. Specifically, for HLA-II molecules, the fused representation of complete sequences of HLA-II chains introduces a more comprehensive knowledge context compared with pseudo sequences. The DSCA mechanism effectively captures key sites, partially mitigating potential noise introduced by full-length sequences. The integration of these two components achieves complementarity, ultimately yielding optimal modeling and predictive performance.

### 2.4 Binding core prediction

In the interaction between peptides and MHC-II molecules, the peptide’s core region typically consists of a contiguous stretch of nine amino acid residues, referred to as the binding core [42]. This region is accommodated within the binding groove of the MHC-II molecule and interacts with HLA-II via key anchor residues, thereby influencing peptide binding and presentation. As illustrated in Fig 9F, DSCA-HLAII is designed to effectively capture the positions of binding cores based on the fused peptide and HLA-II features. The position with the highest score is regarded as the predicted start position of the binding core by DSCA-HLAII.

**Fig 5:**
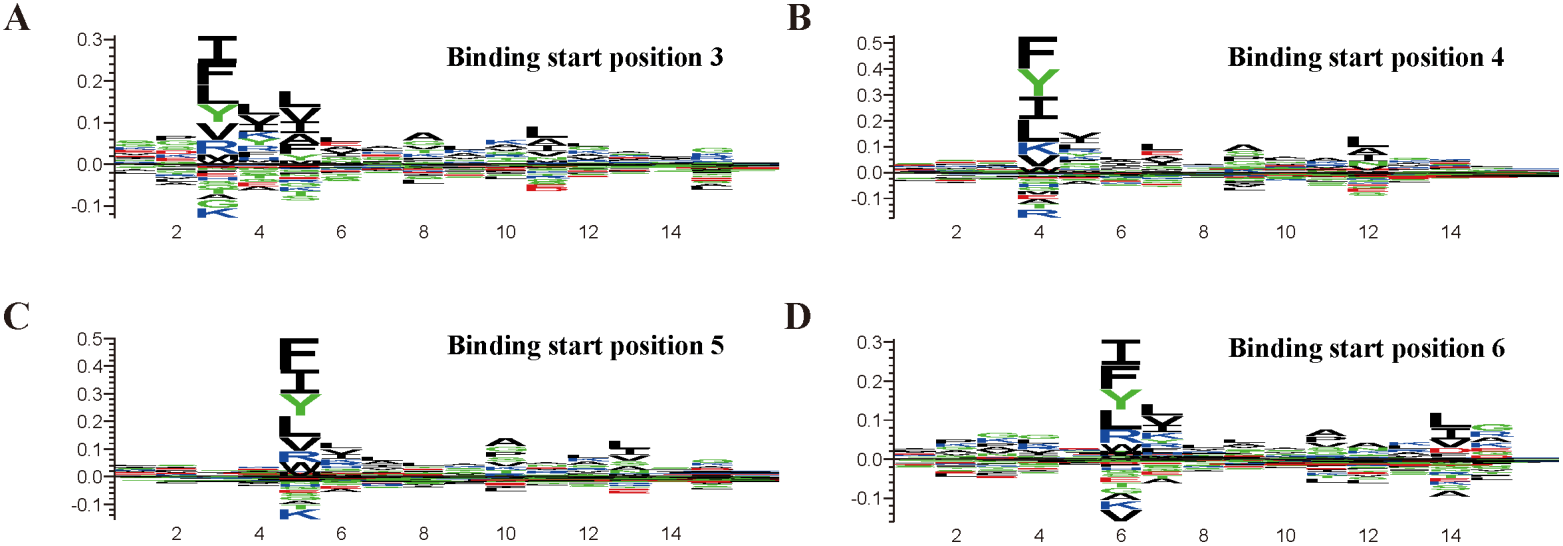
Sequence logos of binding cores at different start positions by DSCA-HLAII. The x-axis denotes residue positions within the peptide. At each position, the total height represents the relative information content, reflecting the degree of conservation, while the height of each letter corresponds to the contribution of the respective amino acid at that position.

**Fig 6:**
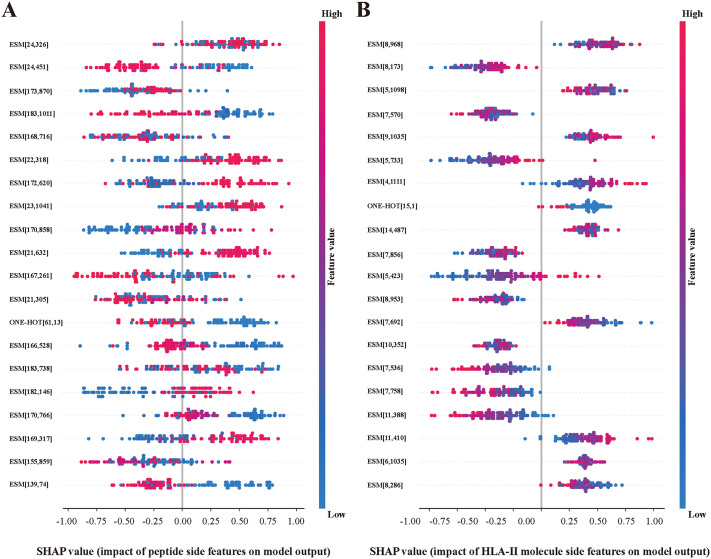
Interpretable Analysis of DSCA-HLAII based on SHAP. (A) The top 20 most influential features of peptide side that impact the model’s predictions, where ESM denotes the ESMC features, ONE-HOT denotes the ONE-HOT features, [*a*, *b*] represents position *a* of the amino acid and position *b* of the feature. (B) The top 20 most influential features of HLA-II molecule side that impact the model’s predictions.

**Fig 7:**
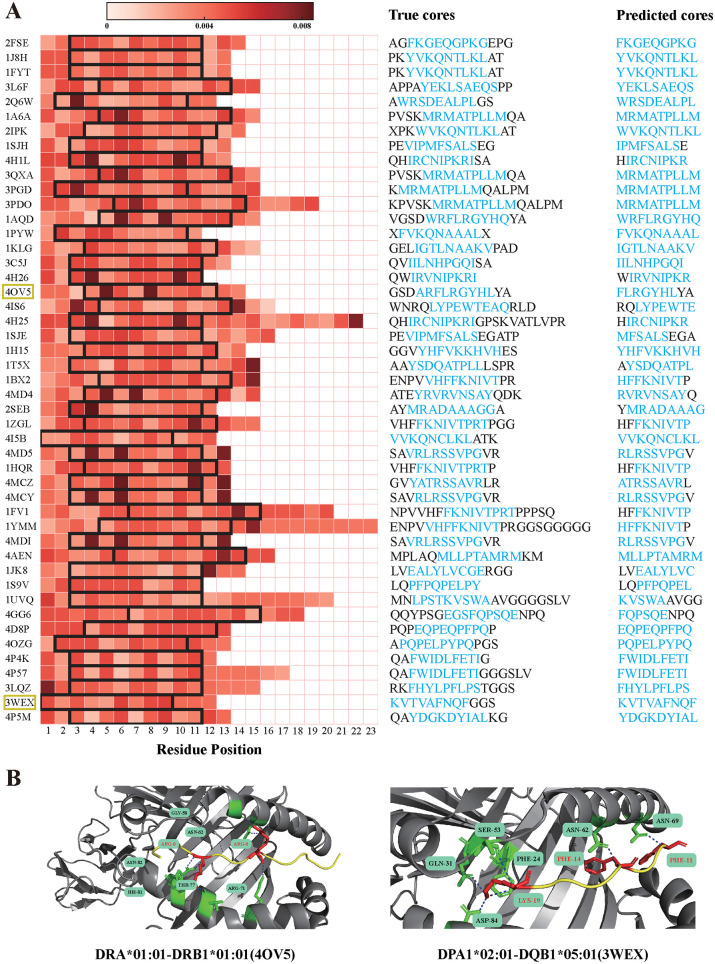
Visualization of DSCA-HLAII average attention weight. (A) Heatmap visualizing the positional attention of peptide sequences within peptide–HLA-II complexes. Each row corresponds to a peptide sequence in a given complex, and each column represents a residue position. The color intensity indicates the magnitude of the attention weight, with black boxes highlighting the binding core. The True cores column on the right annotates each complex with its corresponding peptide sequence and the experimentally validated binding core positions. The Predicted cores column indicates the binding core positions predicted by DSCA-HLAII. (B) Structural visualization of peptide–HLA-II complexes. Peptide sequences are shown in yellow, residues with high attention weights are highlighted in red, polar interactions are indicated in blue, and HLA-II residues interacting with high-attention peptide residues are marked in green.

**Fig 8:**
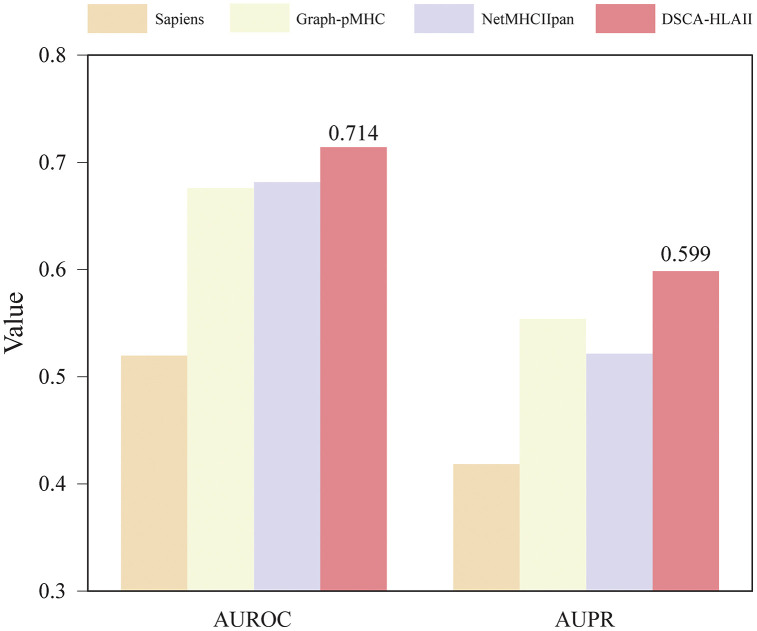
Comparison of the performance of DSCA-HLAII and other methods on the clinical antibody immunogenicity dataset.

**Fig 9:**
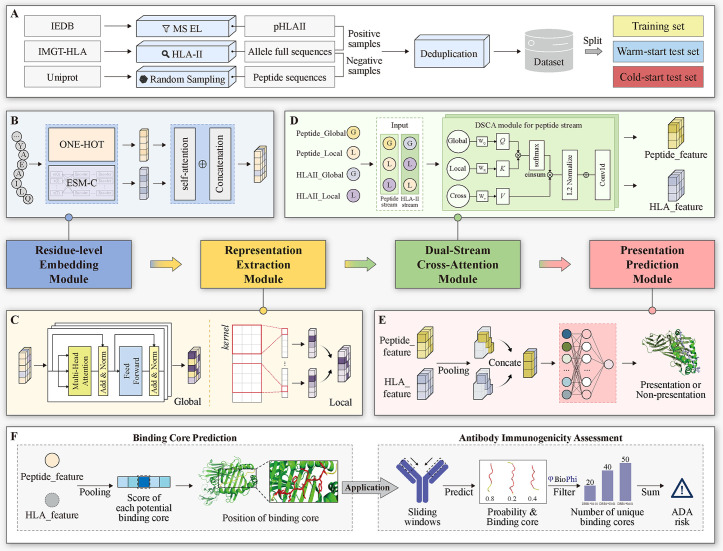
Overview of the DSCA-HLAII framework. A: Data preparation workflow. B: The Residue-level Embedding module. This module extracts ONE-HOT and ESMC representations while incorporating context-enhanced embeddings, providing a comprehensive representation of both peptides and HLA-II molecules. C: The Representation Extraction module. This module captures multi-level dependencies in sequences from global and local perspectives. D: The Cross Attention module. This module employs the DSCA mechanism to capture the information interaction between peptides and HLA-II molecules. The peptide_feature and HLA_feature are constructed through the peptide stream and HLA stream, respectively. E: The Presentation Prediction module. This module outputs the predicted presentation probability based on the integrated interaction features of peptides and HLA-II molecules. F: Downstream Tasks. DSCA-HLAII is used for predicting peptide binding cores and assessing antibody immunogenicity.

The binding cores predicted by DSCA-HLAII provide insights into the potential biological mechanisms underlying the peptide-MHC-II presentation pathway. To validate the effectiveness of binding core prediction, we used the allele DRA*01:01-DRB1*01:01 as an example and visualized peptides corresponding to binding cores at different start positions as sequence logos [43] to examine residue conservation. Specifically, we first employed DSCA-HLAII to predict the presentation probabilities of 100,000 randomly selected peptide segments from the UniProt database. The top 1% of peptides with the highest predicted presentation probabilities were then selected, and sequence logos were generated using Seq2Logo with default settings [44].The results are shown in Fig 5.

As shown in Fig 5, the binding core motifs remain highly conserved regardless of the start position of the binding core. Previous studies have indicated that pocket 1 represents the largest and most critical side-chain binding pocket in HLA-DR1 [45]. Our results demonstrate that the P1 position exhibits the highest relative information content, which further supports the validity of DSCA-HLAII in binding core prediction. Moreover, the P1 position is predominantly enriched with large hydrophobic or aromatic residues such as Phe (F), Tyr (Y), and Ile (I), consistent with previous findings on the pocket binding preferences of HLA-DR molecules [46]. In addition to P1, the P6 and P9 positions were identified by DSCA-HLAII as key anchor residues essential for peptide–HLA-II binding [47]. Therefore, DSCA-HLAII is capable of accurately identifying informative binding cores across different start positions of peptide binding cores.

### 2.5 Interpretability in DSCA-HLAII: effective feature learning and identifying key binding sites

We explore the interpretable insights of DSCA-HLAII from two perspectives: the explainability analysis of input feature importance and the visualization of attention weights from the DSCA mechanism. This interpretability allows for a more detailed, residue-level characterization of the interaction mechanisms between peptides and HLA-II molecules.

#### 2.5.1 Analysis of the importance of multi-view features.

We applied the SHapley Additive exPlanations (SHAP) method [48] to evaluate the importance of different features on both the peptide and HLA-II molecule sides. In the context of peptide–HLA-II interaction prediction, Fig 6A and Fig 6B respectively present the top 20 features contributing most substantially to the DSCA-HLAII model from the peptide side and the HLA-II molecule side. The results indicate that the ESMC features dominate on both sides, highlighting their critical role in the model’s predictive process. This suggests that incorporating pre-trained embeddings facilitates the capture of key semantic information influencing presentation, thereby enhancing prediction accuracy and generalization capability. Notably, on the peptide side, features corresponding to residues 5–8 constitute a large portion of the top 20 features, whereas on the HLA-II molecule side, features near residues 23 and 170 occupy a significant proportion. These findings partially reveal the positional distribution of key sites in peptides and HLA-II molecules that are relevant for antigen presentation.

#### 2.5.2 Residue-level analysis of dual-stream cross-attention.

The DSCA module can compute the attention weight for each site within a peptide sequence, reflecting the relative contribution of individual residues to peptide presentation. We selected peptide-HLA-II pairs from the BC2015 dataset [3] and computed the attention weights for the peptide sequences using DSCA-HLAII, visualizing the distribution of these weights via heatmaps. As shown in Fig 7A, high-attention sites are predominantly concentrated within the binding core region, indicating that the DSCA module effectively highlights key residues relevant to binding and provides meaningful interpretability for subsequent peptide–HLA-II presentation predictions. Furthermore, we compared the binding core positions predicted by DSCA-HLAII with the experimentally validated cores. The results demonstrate that DSCA-HLAII can accurately identify the true binding core regions. Owing to its integration of contextual information from both the peptide sequence and the HLA-II molecular sequence, a small number of predicted cores exhibit a positional shift of 1–2 residues. From the visualized dataset, we further selected representative complexes formed by HLA-DR, HLA-DP, and HLA-DQ molecules and illustrated their PDB structures in Fig 7B. Residues with higher attention weights are generally located within the binding groove of the HLA-II molecules and participate in peptide–HLA-II interactions. For instance, in the HLA-DRA*01:01–DRB1*01:01–peptide complex (PDB ID: 4OV5), the sixth residue of the peptide, ARG, forms a stable hydrogen bond with ASN82 of the HLA β-chain. Similar interaction patterns were observed in other HLA-DP and HLA-DQ complexes, suggesting that the attention weights captured by the model reasonably correspond to structurally critical interacting residues. These findings further demonstrate that the DSCA mechanism can identify the key sites of peptides within HLA-II molecules. In addition, it highlights residues that play crucial roles in molecular interactions, providing structural-level interpretability for the model’s predictions.

### 2.6 Antibody immunogenicity risk assessment

The immunogenicity of an antibody is one of the key indicators for evaluating its potential as a safe and effective therapeutic candidate, as it determines whether the antibody may elicit adverse immune responses in vivo [49]. Previous studies have shown that immunogenicity is closely associated with the recognition of potential T-cell epitopes within the antibody molecule, a process that primarily depends on the binding and presentation of antibody-derived peptide fragments by HLA-II molecules [50]. Therefore, elucidating peptide–HLA-II interactions is of critical importance for predicting antibody immunogenicity risk [51]. In this study, DSCA-HLAII not only accurately predicts the binding affinity between peptides and HLA-II molecules but also facilitates the assessment of antibody immunogenicity risk, thereby providing valuable guidance for the safety evaluation and rational design of antibody therapeutics.

Firstly, we extracted all potential peptide fragments from antibody sequences using sliding windows of lengths 12–19 and predicted their presentation probabilities and binding cores for eight specific HLA-II alleles. Subsequently, peptides whose binding cores appeared in more than 22 subjects were filtered out using the OASis [52] to reduce interference from self-derived peptides. Peptides with presentation probabilities below a defined CUTOFF threshold were then further excluded. Finally, the total number of unique binding cores for each allele was counted and used as an indicator of antibody immunogenicity risk. The eight selected HLA-II alleles in this study correspond to common HLA-DR subtypes, including DRB1*01:01, DRB1*03:01, DRB1*04:01, DRB1*07:01, DRB1*08:01, DRB1*11:01, DRB1*13:01, and DRB1*15:01 [16].

As shown in Fig 8, we compared the performance of DSCA-HLAII with Sapiens [52], Graph-pMHC [16], and NetMHCIIpan [15] on the clinical antibody immunogenicity dataset [16]. The results demonstrate that DSCA-HLAII outperforms the other methods in predicting antibody immunogenicity. This indicates that, in addition to its superior performance in peptide–HLA-II presentation prediction, DSCA-HLAII also possesses strong predictive capability for assessing the immunogenicity risk of antibodies.

## 3. Discussion

This study introduces a novel multi-task predictive framework, DSCA-HLAII, based on a DSCA architecture, which provides a unified approach for modeling peptide–HLA class II interactions, identifying binding cores, and predicting peptide presentation. By integrating ONE-HOT encoded sequence features with pre-trained semantic embeddings (ESMC), the framework constructs a comprehensive hybrid representation of peptide and full-length HLA-II sequences, enabling a thorough encoding of the structural semantics of peptide–HLA-II complexes. Notably, the proposed DSCA module allows for fine-grained, position-aware interaction modeling between peptides and HLA sequences, significantly enhancing the interpretability of critical binding site identification and laying a structural foundation for the model’s generalization capability in cold-start scenarios.

Experimental validation demonstrates that DSCA-HLAII outperforms existing state-of-the-art methods across multiple warm-start and cold-start datasets, highlighting its dual strengths in accuracy and robustness. The model simultaneously predicts peptide presentation probabilities and binding core locations while supporting systematic assessment of antibody immunogenicity risk, thereby offering a novel computational tool for vaccine design and immunotherapeutic research. To promote practical application, we have further developed a publicly accessible web server (http://bliulab.net/DSCA-HLAII), lowering the barrier to adoption for researchers in the field.

In summary, DSCA-HLAII provides a more accurate, interpretable, and practical solution for predicting peptide–HLA class II interactions by integrating multi-source sequence features with attention-driven interaction modeling. While the model has demonstrated superior performance across multiple test sets, future work may further explore its predictive capacity for rare alleles, incorporate structural information to enrich representations, and deepen its application in peptide vaccine design and personalized immunotherapy. The framework proposed in this study also offers a transferable modeling strategy for other protein–ligand interaction prediction tasks.

## 4. Conclusion

In this study, we propose DSCA-HLAII, a novel predictive model for peptide–HLA-II interactions and presentation. The model systematically integrates pre-trained semantic and structural ESMC information and ONE-HOT to construct multi-view representations. It then leverages a specially designed DSCA to enable comprehensive learning and effective capture of interaction patterns between peptides and HLA-II molecules. Extensive experimental evaluations demonstrate that DSCA-HLAII consistently achieves state-of-the-art predictive performance and robust generalization capability, while simultaneously offering valuable biological interpretability. Moreover, the model can be extended to binding core prediction and antibody immunogenicity assessment, where it likewise exhibits superior performance. Finally, we have developed an online server, which is publicly accessible at http://bliulab.net/DSCA-HLAII.

Although DSCA-HLAII demonstrates substantial predictive performance, several limitations remain. Our approach only considers single-allelic (SA) samples while neglecting multi-allelic (MA) samples that contain multiple alleles. As a result, DSCA-HLAII may miss learning allele-specific information present exclusively in MA samples. Additionally, because the model incorporates contextual information, while it enhances the modeling of peptides and HLA-II molecules, it may also introduce slight deviations in accurately identifying the precise positions of binding cores. In future work, we aim to integrate MA and SA data more effectively to further enhance the model’s generalization capability. Some recent advanced studies have introduced graph-based learning strategies to recognize and model protein complexes, such as FMvPCI [53] and LCAAG [54]. We plan to achieve a comprehensive improvement in both the modeling of peptide–HLA-II interactions and the accurate identification of key sites through more advanced graph learning mechanisms.

## 5. Methods

### 5.1 Benchmark and independent test datasets

For the benchmark dataset, the data were systematically curated from multiple authoritative sources, including the IEDB [55], UniProt [56], and IPD-IMGT/HLA [57] databases. For positive peptide–HLA-II pairs, we selected data annotated with the mass spectrometry eluted ligand (MS EL) label from IEDB, as this label reflects the processes of antigen processing and presentation and is generated under more standardized experimental conditions. To obtain complete sequences of HLA-II chains corresponding to the positive pairs, the complete *α* chain and *β* chain sequences of each allele were retrieved from the IPD-IMGT database. Based on the records in the UniProt database, the *α* chain and *β* chain sequences of different alleles were then segmented at specific positions and concatenated to construct the complete sequences of HLA-II chains for each allele.

For negative peptide–HLA-II pairs, peptide sequences with lengths ranging from 8 to 32 amino acids were randomly extracted from the UniProt database to generate a peptide pool of two million sequences. The complete sequences of HLA-II chains were constructed using the same strategy as for the positive pairs. To enhance the model’s generalization capability, we constructed a negative-to-positive sample ratio of 5:1 for each allele. When the available negative samples for a particular allele did not suffice to meet this ratio, additional negative instances were produced by randomly sampling peptides from the peptide pool and pairing them with that allele. To ensure that the peptide length distributions of the positive and negative samples remained consistent, we enforced that the peptide lengths of the newly generated negative samples matched those of the positive samples for each allele.

To rigorously evaluate model performance, all peptide sequences that appeared in the independent test dataset were removed from the benchmark dataset, rather than only excluding peptide–allele pairs with exact matches. This deduplication procedure effectively prevents potential information leakage caused by repeated peptide sequences. Finally, the benchmark dataset comprised 76 HLA-II alleles and a total of 942,602 peptide–HLA-II pairs, including 154,324 positives and 788,278 negatives.

For the independent test dataset, we constructed warm-start and cold-start test datasets based on allele coverage [18]. Alleles in the warm-start test dataset had appeared in the benchmark dataset, whereas alleles in the cold-start test dataset were entirely absent from the benchmark dataset. The warm-start test dataset ultimately included 76 HLA-II alleles and comprised a total of 192,846 peptide–HLA-II pairs, of which 32,141 were positive samples and 160,705 were negative samples. The cold-start test dataset contained 55 HLA-II alleles and a total of 5,262 peptide–HLA-II pairs, including 877 positive and 4,385 negative samples.

### 5.2 The architecture of the DSCA-HLAII

The framework of DSCA-HLAII is illustrated in Fig 9. It consists of four main stages: Residue-level Embedding, Representation Extraction, Cross-Attention, and Presentation Prediction. This framework is designed to extract both global and local features enriched with semantic information from peptide and HLA-II sequences. By incorporating the DSCA mechanism, the model explicitly captures the dependencies between the two sequences. This design enables the prediction of presentation probabilities and supports downstream tasks such as binding core identification.

In the Residue-level Embedding stage, peptides and HLA-II molecules are represented from two perspectives: sequence-based features and pre-trained embeddings. Specifically, sequence features are encoded using ONE-HOT representations to capture residue-level information. ESMC embeddings encode semantic, structural, and functional information of the sequences. During the Representation Extraction stage, sequence information is extracted from both global and local perspectives. Global features are captured through several layers of transformer encoders and local features are obtained via one-dimensional convolution operations that downsample the peptides and HLA-II sequences. In the Cross-Attention stage, the DSCA mechanism is employed. This mechanism not only captures key sites information within peptide and HLA-II sequences but also explicitly models the dependencies between the peptide and HLA-II sequences. In the Presentation Prediction stage, the fused peptide–HLA-II representations are used to predict presentation probabilities via a binary cross-entropy (BCE) loss function. Moreover, DSCA-HLAII can be extended to downstream tasks, including binding core prediction and antibody immunogenicity assessment, demonstrating the potential utility of our approach in immunological research.

### 5.3 Residue-level embedding module

The Residue-level Embedding module directly determines whether the model can effectively utilize the relevant inputs of peptides and HLA-II molecules, thereby influencing the predictive capability and accuracy of downstream tasks. In this study, DSCA-HLAII extracts both the sequence-based ONE-HOT encoding and the pretrained semantic ESMC representation of peptides and HLA-II molecules. The ONE-HOT encoding reflects residue-level information and preserves fundamental sequence features [58,59]. The ESMC representation captures deeper semantic information, revealing the contextual relationships across amino acids and encoding potential structural and functional characteristics [39]. The sequences of peptides and HLA-II molecules can be represented as $S_{pep}=\{R_{\text{1}}R_{\text{2}}R_{\text{3}}\ldots R_{L_{pep}}\}$ and $S_{hla}=\{R_{\text{1}}R_{\text{2}}R_{\text{3}}\ldots R_{L_{hla}}\}$, where $R_{i}$ denotes one of the standard amino acids or the non-standard amino acid ‘X’, $L_{pep}$ represents the length of the peptide, and $L_{hla}$ refers to the length of the concatenated *α* chains and *β* chains of the HLA-II molecule.

ESMC, a widely used protein language model from the ESM3 family, is purpose-built for advancing protein representation learning [39]. By automatically extracting deep biological features, it avoids the need for the supervisory sequences and feature engineering that traditional methods rely on. ESMC achieves significant gains in predictive performance alongside enhanced computational efficiency. In this study, we employed the ESMC-600M model to extract features from peptide sequences and the full-length chain sequences of HLA-II molecules. After processing by the ESMC model, peptide sequences $S_{pep}$ and HLA-II sequences $S_{hla}$ are represented as vectors of dimensions ${(L}_{pep}*\text{1}\text{1}\text{5}\text{2})$ and ${(L}_{hla}*\text{1}\text{1}\text{5}\text{2})$, respectively, as defined in the following equation:


$$\begin{array}{c} E_{pep}=ESM\left(S_{pep}\right) \end{array}$$ (1)

$$\begin{array}{c} E_{hla}=\left[ESM\left(S_{hla_{alpha}}\right);ESM\left(S_{hla_{beta}}\right)\right] \end{array}$$ (2)

where $S_{hla\_alpha}$ and $S_{hla\_beta}$ denote the *α* chain and *β* chain of the HLA-II molecule, respectively. Due to the heterodimeric nature of HLA-II molecules, the *α* chains and *β* chains of $S_{hla}$ are processed independently. Specifically, each chain is separately fed into the ESMC model to generate its representation, and the two representations are then concatenated to construct the comprehensive representation of $S_{hla}$ using Eq.(2).

The ONE-HOT feature encodes the sequence into a high-dimensional, sparse binary vector representation. Since the lengths of peptide sequences are inherently heterogeneous, we standardize the sequences to a fixed length of 32 through padding or truncation. Consequently, the peptide sequence $S_{pep}$ and the HLA-II molecular sequence $S_{hla}$ are represented as (32*21)-D and (200*21)-D vectors. In addition, we design a self-attention layer to extract context-enhanced representations from the ONE-HOT, enabling the capture of context-dependent relationships across residues within both the peptide and the complete sequences of HLA-II chains. By aligning and concatenating the two types of representations in a latent space, we obtain the fused features of the peptide and HLA-II molecule, denoted as $E_{multi}$:


$$\begin{array}{c} E_{multi}=\left[E_{esm};E_{one-hot};SelfAttn\left(E_{one-hot}\right)\right] \end{array}$$ (3)

where $E_{esm}$ and $E_{one-hot}$ denote the features of ESMC and ONE-HOT, respectively. SelfAttn() denotes the self-attention layer.

Therefore, the fused feature representation comprises both ONE-HOT and ESMC features. The ONE-HOT representation captures the fundamental sequence features, while the ESMC representation encodes deeper semantic information, reflecting contextual dependencies and latent relationships within the sequence. The fused representation provides the model with comprehensive and informative input features, enriching the representational space of peptides and HLA-II molecules.

### 5.4 Representation extraction module

The model is designed with a feature extraction framework that integrates both global and local encoding strategies to fully capture the multi-level dependencies in features. The synergy between these two strategies allows the model to balance long-range structural information with local functional site information, thereby enhancing both prediction accuracy and generalization capability.

To capture global contextual dependencies, we designed a Transformer-based encoding module, consisting of a stack of three Transformer encoder layers. Formally, each Transformer encoder layer updates the input ${𝐗}^{\left(l\right)}\in R^{B\times L\times D}$ by applying multi-head self-attention (MHSA) followed by a position-wise feed-forward network (FFN), with residual connections and layer normalization:


$$\begin{array}{c} {𝐙}^{\left(l\right)}=\text{LayerNorm}\left({𝐗}^{\left(l\right)}+\text{MHSA}\left({𝐗}^{\left(l\right)}\right)\right) \end{array}$$ (4)

$$\begin{array}{c} {𝐗}^{\left(l+\text{1}\right)}=\text{LayerNorm}\left({𝐙}^{\left(l\right)}+\text{FFN}\left({𝐙}^{\left(l\right)}\right)\right) \end{array}$$ (5)

where ${𝐗}^{\left(l\right)}$ denotes the input to the l-th Transformer encoder layer, and ${𝐙}^{\left(l\right)}$ represents the intermediate representation at the same layer. Through the global encoding process, the model is able to capture dependencies within the multi-source fused features. Compared with standard self-attention, the MHSA mechanism allows multiple queries to focus on different relational patterns within the sequence, thereby capturing comprehensive information.

To capture local short-range dependencies, we apply one-dimensional convolution operations to downsample the features. The design of the convolutional kernels incorporates biological prior knowledge of both peptides and HLA-II molecules. For peptide sequences, considering the length of the binding core, the kernel size is set to 9 to effectively capture local information for each potential binding core. For HLA-II sequences, taking into account the positional distribution of key binding pockets, the kernel size is set to 5 to facilitate the capture of contextual relationships among residues within local pockets. Finally, the resulting local short-range dependency features are denoted as $H_{local}$. This process can be formally represented as:


$$\begin{array}{c} H_{local}=Conv\text{1}D\left(E_{multi}\right) \end{array}$$ (6)

Therefore, the global encoding is designed to capture contextual associations across the entire sequence, enabling the characterization of both overall structural features and long-range interactions. In contrast, the local encoding targets fine-grained features by incorporating biological prior knowledge into the design of convolutional kernels, which enables the effective capture of local dependencies around the binding core and key pocket regions.

### 5.5 Dual-stream cross-attention module

In this section, we propose the DSCA model to precisely capture the interaction between the peptide and HLA-II sequences. In conventional cross-attention mechanisms, the Query is typically derived from the peptide sequence, whereas the Key and Value originate from the HLA-II sequence [60]. This design ensures a unidirectional information flow, where features are queried and fused exclusively from the peptide to the HLA-II molecule. However, peptides and HLA-II molecules influence each other during the actual interaction process. The conventional cross-attention mechanism based on the unidirectional interaction may be insufficient to fully characterize their interaction process. Moreover, the attention computation for each position of the peptide and HLA-II molecule is homogeneous, which may hinder the identification of key sites. To address the above limitations, we propose the DSCA module, which operates by processing the global and local features of the peptide and the HLA-II molecule through Peptide stream and HLA-II stream. Through the Peptide stream and HLA-II stream dual-stream design, the interaction process between the peptide and the HLA-II molecule can be more comprehensively modeled. Using the peptide stream as an illustrative example, the operational pipeline of the DSCA module can be described as follows.

Initially, in the Peptide stream, the global feature representation of the peptide is mapped via a linear transformation into the Query space, while the local feature representation of the peptide is mapped via a linear transformation into the Key space. The local feature representation of the HLA-II serves as the Cross information and is mapped via a linear transformation into the Value space. This process can be formally represented as:


$$\begin{array}{c} Q_{cross}=X_{pep\_global}W_{Q},K_{cross}=X_{pep\_local}W_{K},V_{cross}=Y_{HLA\_local}W_{V} \end{array}$$ (7)

where $X_{pep\_global}$ denotes the global feature of the peptide, $X_{local}$ denotes the local feature of the peptide, and $Y_{HLA\_local}$ denotes the local feature from the HLA-II.

Subsequently, the attention scores, computed from the Query and Key, are then leveraged to aggregate the Value via the Einstein summation convention [61], producing preliminary cross-fused information. The resulting representation is subsequently normalized to ensure a stable feature distribution. Notably, the dimensions of the cross-fused information are aligned with the sequence positions, such that the values of the fused representation partially reflect the distribution of key sites.


$$\begin{array}{c} Attn=\text{Softmax}\left(\frac{Q_{cross}{K_{cross}}^{T}}{\sqrt{d_{pep\_global}}}\right) \end{array}$$ (8)

$$\begin{array}{c} O=Attn\cdot V_{cross} \end{array}$$ (9)

$$\begin{array}{c} \tilde{O}={\left\|O\right\|}_{\text{2}} \end{array}$$ (10)

where $d_{pep\_global}$ denotes the dimensionality of the global feature in the peptide stream.

Finally, to capture potential key regions, a one-dimensional convolution, using the same parameters as in the encoding stage, is applied to the fused representation. The result is then combined with the input global features via a residual connection to obtain the final peptide_feature representation $H_{pep}$. On the peptide side, the dimensions of the final feature representation are aligned with the potential start positions of the binding core, such that the values of the final representation further reflect the distribution of key sites.


$$\begin{array}{c} H_{pep}=Conv\text{1}D\left(\tilde{O}\right)+X_{pep\_global} \end{array}$$ (11)

The Query, derived from the global feature, represents the overall semantics of the entire sequence. The Key, derived from the local feature, captures fine-grained information for each segment. The global feature queries the local feature, directing the attention to key local positions that are most relevant for interaction and reducing the impact of irrelevant positions. This mechanism is utilized to capture contextual dependencies. The Value, derived from the local feature of the other sequence, facilitates cross-stream information transfer. Specifically, the Query–Key attention first identifies key sites within the current sequence, and then the Value extracts the corresponding key sites from the other sequence. This process is consistent with the biological principles of peptide–HLA-II interactions and improves the model’s generalization performance.

Similarly, in the HLA-II stream, the DSCA module follows the same workflow as described for the peptide stream, which generates the final HLA_feature $H_{HLA}$. In summary, the DSCA module, through its dual-stream and cross-stream attention design, enables precise modeling of the bidirectional interactions between peptides and HLA-II molecules. The Query–Key–Value construction based on global and local features not only enhances the identification of key sites but also improves the generalization performance of the model.

### 5.6 Presentation prediction

The Presentation Prediction module predicts the presentation probability by integrating the interaction features of peptides and HLA-II molecules. Firstly, the peptide feature $H_{pep}$ and HLA-II feature $H_{HLA}$ are separately subjected to global max pooling and concatenation to obtain a joint interaction representation of the peptide and HLA-II, denoted as $G_{interaction}$. Next, a MLP module followed by a linear projection and a sigmoid activation function is used to generate the presentation probability $\hat{y}$, represented as:


$$\begin{array}{c} \hat{y}=\sigma \left(W\cdot \text{MLP}\left(G_{interaction}\right)+b\right) \end{array}$$ (12)

where W and b represent the weight and bias of the final linear projection layer, and σ denotes the sigmoid activation function.

Given that peptide–HLA-II presentation prediction constitutes an imbalanced binary classification problem, the model is trained using the BCELoss function [62], which effectively quantifies the discrepancy between predicted probabilities and true labels, thereby improving the model’s discriminative performance between positive and negative classes. It is formally defined as follows:


$$\begin{array}{c} l_{BCE}=-\frac{\text{1}}{N}\sum_{i=\text{1}}^{N}\left[y_{i}\;\text{log}\left({\hat{y}}_{i}\right)+\left(\text{1}-y_{i}\right)\text{log}\left(\text{1}-{\hat{y}}_{i}\right)\right] \end{array}$$ (13)

where N denotes the number of samples, ${\left\{y_{i}\right\}}_{i=\text{1}}^{N}\in \{\text{0},\text{1}\}$ represents the ground-truth label of the i-th sample, and ${\left\{{\hat{y}}_{i}\right\}}_{i=\text{1}}^{N}\in [\text{0},\text{1}]$ denotes the predicted probability of the i-th sample being positive.

### 5.7 Implementation and training

We implemented DSCA-HLAII using the PyTorch framework (https://pytorch.org/). The model was trained with the Adadelta optimization algorithm [63] for parameter optimization. The learning rate for model training was set to 0.01. To enhance model generalization, we incorporated several key techniques: (1) Dropout regularization to prevent overfitting [64], (2) Early stopping based on validation performance to optimize training duration [65]. The patience parameter for early stopping was set to 7, (3) A weight decay strategy was employed to regularize the model parameters [66], with the weight decay coefficient set to 1 × 10^-4^.

To ensure reproducibility and effective performance, these hyperparameters were selected and fine-tuned using 5-fold cross-validation on the benchmark set. Specifically, we explored a range of values across the folds and chose the combination that yielded the best average validation performance.

### 5.8 Performance evaluation

The presentation probability prediction task is formulated as a binary classification problem. Therefore, we evaluate the model’s performance using seven metrics: Precision, Recall, MCC, ACC, F1, AUROC, AUPR, and PCC. AUROC measures [67–71] the overall discriminative ability of a model in distinguishing positive and negative samples [72,73]. AUPR assesses model performance by integrating precision and recall trade-offs [74].

Assume the test set contains N samples, each consisting of a peptide–HLA-II pair. The ground-truth label is denoted as ${\left\{y_{i}\right\}}_{i=\text{1}}^{N}\in \{\text{0},\text{1}\}$, and the model’s predicted score is represented as {${\left\{{\hat{s}}_{i}\right\}}_{i=\text{1}}^{N}\in [\text{0},\text{1}]$. The predicted label ${\hat{y}}_{i}\in \{\text{0},\text{1}\}$ is then obtained by applying a threshold τ. The definitions of each metric are as follows [3,75]:


$$\begin{array}{c} \begin{array}{c} \left\{ \begin{array}{c} \text{Precision}=\frac{TP}{TP+FP}\\ {}[5pt]Recall=\;\frac{TP}{TP+FN}\\ {}[5pt]\text{MCC}=\frac{TP\cdot TN-FP\cdot FN}{\sqrt{\left(TP+FP)(TP+FN)(TN+FP)(TN+FN\right)}}\\ {}[8pt]\text{F1}=\frac{\text{2}\cdot \text{Precision}\cdot \text{Recall}}{\text{Precision}+\text{Recall}}\\ {}[5pt]\text{ACC}=\frac{TP+TN}{TP+TN+FP+FN}\\ {}[5pt]\text{PCC}=\frac{\sum_{i=\text{1}}^{N}\left(y_{i}-\overset{―}{y}\right)\left({\hat{s}}_{i}-\overset{―}{\hat{s}}\right)}{\sqrt{\sum_{i=\text{1}}^{N}{\left(y_{i}-\overset{―}{y}\right)}^{\text{2}}}\sqrt{\sum_{i=\text{1}}^{N}{\left({\hat{s}}_{i}-\overset{―}{\hat{s}}\right)}^{\text{2}}}} \end{array}\right.\; \end{array} \end{array}$$ (14)

where *TP* is the true positive, *TN* is the true negative, *FN* is the false negative, *FP* is the false positive, Precision is the proportion of predicted positives that are actually positive, Recall is the proportion of actual positives that are correctly predicted.
